# Inequality in preconception care utilization based on the opportunity inequality framework: evidence from explained machine learning

**DOI:** 10.3389/fpubh.2026.1732192

**Published:** 2026-05-07

**Authors:** Xiaodong Wu, Hai Fang, Tingting Xu

**Affiliations:** 1 School of Public Health, Capital Medical University, Beijing, China; 2 School of Public Health, Peking University, Beijing, China; 3 Institute for Global Health and Development, Peking University, Beijing, China

**Keywords:** preconception care, health equality, machine learning, SHAP, free health service

## Abstract

**Background:**

Despite the universal availability of free preconception care (PCC) in China, substantial disparities in utilization persist. It remains unclear how much of this reflects structural barriers versus individual shortfalls, and clarifying this balance is critical for designing equitable, accountable maternal health policy.

**Methods:**

We conducted a multi-center cross-sectional survey across ten medical facilities in China from July 1, 2023, to July 1, 2024, including 8,866 pregnant women. Guided by the Equality of Opportunity framework, we applied logistic regression to assess PCC factors and three machine learning models to assess factors’ contributions of PCC utilization. Shapley Additive Explanations (SHAP) decomposition quantified the contribution of individual-level “effort” versus structural “circumstance” factors. Subgroup analyses explored heterogeneity by education level, urban–rural residents, whether or not the annual PCC promotion was accepted at local MCH institutions, and pregnancy intention status.

**Results:**

Overall PCC uptake was 42.4%, with disparities strongly associated with individual-level factors as well as structural determinants. Effort-related variables, particularly pregnancy intention and knowledge of PCC, were the strongest predictors across all models, accounting for 74.1–93.6% of explained variation. However, circumstance factors, including annual PCC promotion at local maternal and child health institutions, remained influential, especially among vulnerable subgroups such as women with unplanned pregnancies, for whom structural factors explained nearly half of the disparity.

**Conclusion:**

Our findings reveal that even in systems offering universal coverage, access to PCC remains stratified by structural opportunity. Although individual-level knowledge and intention are key levers, they are themselves shaped by broader social conditions. Addressing PCC inequality thus requires integrated strategies that promote both individual empowerment and systemic equity.

## Introduction

Health inequities remain a persistent challenge in public health. The World Health Organization defines them as avoidable and unjust differences in health outcomes between social groups, largely shaped by structural factors such as income, education, and living conditions (1). Although health inequality has been widely studied across chronic disease, maternal health, and healthcare access, important gaps remain for vulnerable and understudied populations, including women in the preconception period, those living in rural or remote areas, and those experiencing unplanned pregnancies (2–4).

Among these underexamined areas, preconception care (PCC) stands out as both clinically essential and analytically neglected. PCC encompasses biomedical, behavioral, and social interventions provided before conception to reduce preventable maternal and neonatal risks (4, 5). Increasingly recognized as a cornerstone of reproductive health and gender equity, PCC has gained global policy attention (5–9). However, despite its preventive purpose and potential to mitigate long-term risks, the equity dimensions of PCC utilization remain poorly understood.

Evidence from diverse settings consistently shows that PCC uptake is highly stratified by socioeconomic status, education, and geography. While such patterns have been observed internationally (10, 11), few studies have systematically investigated PCC inequities in rapidly developing contexts where health services are widely available but disparities in utilization persist. Understanding these dynamics is critical to determining whether, and how, formal access translates into equitable health opportunities in transitioning societies.

China offers a particularly relevant context for this inquiry. Since 2010, the Chinese government has implemented a nationwide, publicly funded PCC program offering free services to all eligible couples (12, 13). While reflecting a strong national commitment to maternal and child health, uptake remains below 50% in many provinces, with pronounced disparities by urban–rural residence, socioeconomic status, and pregnancy intention (14). These persistent inequities, despite universal service provision, underscore the complex interplay between structural disadvantage and individual agency. They also highlight the importance of distinguishing disparities arising from modifiable personal behaviors and those rooted in systemic barriers. In China, research on PCC has developed relatively late and remains limited in scope. Most existing studies have focused on the content and effectiveness of PCC services, followed by explorations of service patterns. Only a small number of studies have examined PCC utilization, and these have largely concentrated on identifying associated factors rather than assessing the normative implications of inequality (15, 16). As a result, it remains unclear to what extent the observed disparities reflect differences in individual behaviors and preferences, and to what extent they arise from structural disadvantage. This distinction is particularly important, because policy may differ substantially depending on whether inequalities are driven by modifiable individual factors or by external circumstances beyond personal control.

In this study, Equality of Opportunity (EOp) theory, also referred to as Roemer’s theory, was used as the normative framework for interpreting fair and unfair inequalities in PCC utilization (17). In this framework, inequalities arising from circumstances are considered unfair because circumstances refer to factors beyond individual control, such as socioeconomic background, and other structural conditions that shape the opportunities available to individuals. By contrast, inequalities related to effort are considered, at least normatively, to reflect differences in individual choices, actions, or behavioral responses for which individuals may be held responsible. Following this framework, the variables in this study were classified into circumstance and effort domains according to their conceptual relationship to individual responsibility and control. Variables reflecting social and economic conditions and broader contextual constraints were assigned to the circumstance domain, whereas variables more closely related to individual health-related behaviors and decision-making were assigned to the effort domain. We acknowledge that the classification of some variables may be context-sensitive and involve normative judgment, as recognized in the broader EOp and health inequality literature. While EOp has been widely applied in economics and education research, its use in health services, particularly maternal and reproductive health, remains limited. Given the coexistence of universal service provision and persistent social gradients in China, the EOp framework provides a particularly relevant lens for analyzing inequities in this context.

Therefore, this study aimed to address two questions: whether inequality exists in PCC utilization among Chinese women, and to what extent such inequality is associated with circumstance-related versus effort-related factors within an EOp framework. We hypothesized that PCC utilization would be unequally distributed and that circumstance-related factors would account for a substantial share of the observed inequality.

## Methods

### Study design and setting

This cross-sectional study was conducted between July 1, 2023, and July 1, 2024 across ten healthcare facilities located in eight provinces in China. Provinces selected in this study were showed in Supplementary Figure 1. Healthcare facilities were selected using purposive (maximum-variation) sampling to capture diversity in geographic region, economic development level, and urban–rural context. A total of 10 healthcare institutions from 8 provinces were included, with 1–2 institutions selected per province. Facility selection also considered feasibility factors, including institutional willingness to participate and capacity to support data collection. The selected facilities collectively provide antenatal care and delivery services for approximately 80% of pregnant women within their respective catchment areas. Given that over 95% of births in China occur in medical institutions (18), this site selection strategy was intended to ensure that the study sample closely reflects the underlying pregnant population in each locality. While the facilities were not randomly selected, their inclusion was designed to enhance heterogeneity and support the generalizability of findings across diverse contexts. Ethical approval was obtained from the Institutional Review Board of Capital Medical University (Z2023SY087).

### Participants, sampling and quality control

Pregnant women receiving antenatal care at the participating healthcare facilities were eligible if they had continuously resided in the local area for at least six months. Women diagnosed with psychiatric disorders or cognitive impairments that could interfere with informed consent or participation were excluded. At each facility, all eligible pregnant women attending antenatal care during the study period were invited to participate. A consecutive recruitment approach was applied to achieve complete enrollment of the target population within each site and time frame. All participants provided written informed consent prior to enrollment. Given that this study was designed as a multicenter cross-sectional survey with consecutive recruitment of all eligible women attending the selected facilities during the study period, no formal *a priori* sample size calculation was performed. Several quality-control measures were applied during questionnaire design and administration. Structured response options were used for selected items, such as categorized income levels, and explicit lists of health conditions were provided to reduce misreporting. During data collection, at least one trained healthcare professional at each participating facility was available to provide standardized guidance and answer participants’ questions. All survey staff received unified training from the research team prior to data collection.

### Data collection procedures

Data were collected through structured, face-to-face interviews conducted by trained healthcare professionals at each participating facility. The survey instrument gathered information on: sociodemographic and socioeconomic characteristics (age, education, income, urban–rural), self-rated health status and chronic health conditions, reproductive history and pregnancy intention, knowledge and attitude of PCC, health behaviors and healthcare access, utilization of PCC services. The questionnaire was developed based on national PCC guidelines, prior research, and formative interviews with healthcare providers and pregnant women from diverse regions. The final instrument underwent cognitive testing and expert review to ensure content validity and contextual relevance. Interviewers received standardized training, and data quality was monitored through real-time consistency checks and periodic audits. Item-level missing data were minimal (<5% across variables). Given the use of a full population-based dataset collected across multiple sites, no formal *a priori* sample size calculation was deemed necessary.

### Outcome

The primary outcome was PCC utilization. To address potential variability in participants’ understanding of PCC and minimize recall bias, utilization was assessed using three specific questions referring to the year prior to the current pregnancy: Have you undergone preconception risk assessment, including preconception counseling and training? Have you received preconception health guidance, including guidance on lifestyle and nutrition? Have you had preconception health examinations, such as blood tests or gynecological examinations? Participants who responded “yes” to any of the three questions were classified as PCC users. This composite outcome definition was based on the standard services outlined in China’s National Free Preconception Health Examination Project and aligned with international PCC guidelines (13, 19).

### Independent variables and theoretical framework

Explanatory variables were classified according to Roemer’s EOp framework, which distinguishes between inequalities arising from individual behaviors (effort) and those attributable to structural or contextual factors beyond individual control (circumstances). This distinction was applied to differentiate ethically neutral variations from those warranting policy intervention in the context of PCC utilization.

Circumstance variables reflected broader socioeconomic and environmental conditions shaping access to care and included maternal income, educational attainment, census registration (urban–rural), regions (by economic development level), distance to nearest maternal and child health (MCH) institution, annual PCC promotion at local MCH institutions, and access to annual physical examination.

Effort variables captured individual characteristics and choices plausibly influencing service uptake. These included maternal age, parity, knowledge of PCC (knowledge of PCC services and knowledge of PCC policy), health status (common infectious diseases, chronic diseases and other diseases; adverse pregnancy outcomes and reproductive system diseases), pregnancy intention and attitude toward PCC.

Variable selection and classification were guided by previous research on maternal health inequities and adapted to the contextual realities of China’s healthcare system. Detailed definitions and coding schemes are provided in Supplementary Table 1.

### Statistical analysis

Descriptive statistics were used to characterize the sample. Variables were summarized as frequencies and percentages.

**Figure 1:**
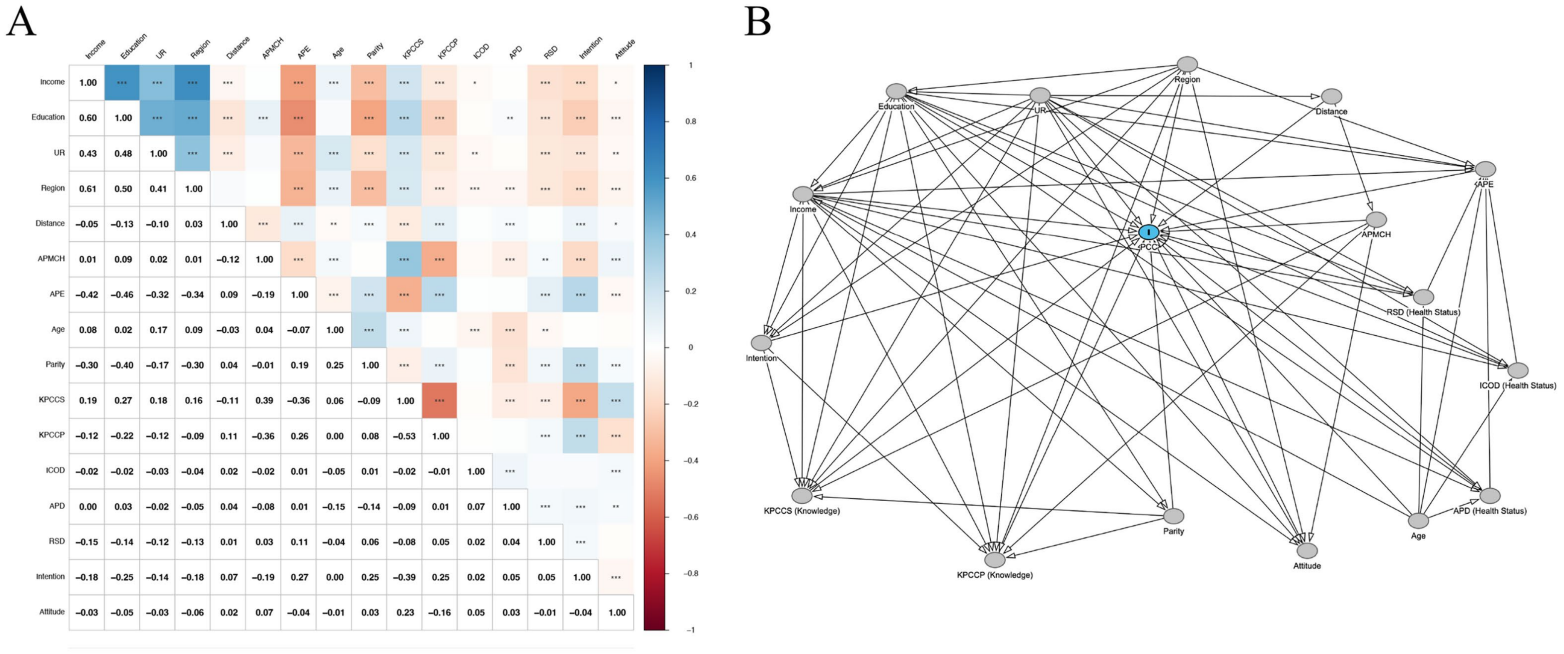
**(A)** Spearman rank correlation analysis for variables. **(B)** Directed acyclic graph. UR, census registration; Distance, distance to nearest maternal and child health institution; AMPCH, annual PCC promotion at local MCH institutions; APE, annual physical examination, KPCCS, knowledge of PCC services; KPCCP, knowledge of PCC policy; ICOD, common infectious diseases, chronic diseases, and other diseases; APD, adverse pregnancy outcomes; RSD, reproductive system diseases; Intention, pregnancy intention, Attitude: attitude toward PCC.

Spearman correlation analysis was performed across all explanatory variables to detect potential overlap between variables (Figure 1A). A directed acyclic graph (DAG), constructed from prior literature and theoretical frameworks, was used to map hypothesized causal relationships (Figure 1B). A detailed account of the decision for each possible causal connection is provided in Supplementary Table 2. In cases where correlation and DAG both suggested dependency, residualization was applied to preserve interpretability and reduce attribution bias. Details of the residualization procedures can be found in Supplementary Text 1.

Logistic regression was used to estimate associations between each explanatory variable and PCC utilization, with results presented as adjusted odds ratios (ORs) and 95% confidence intervals. Univariable logistic regression models were first fitted to estimate the crude association between each variable and PCC utilization. Then, the variables were residualized based on the DAG and Spearman correlation. Univariable logistic regression models were subsequently fitted using the residualized variables to estimate their adjusted associations with PCC utilization. This approach achieved adjustment for confounding among explanatory variables. All statistical tests were two-sided and *p*-values <0.05 were considered significant.

To assess variable importance and decompose contributions to PCC inequality, we applied three supervised machine learning algorithms (logistic classifier, RandomForest, and XGBoost) using the same set of processed variables. Logistic classifier captured linear associations, while the two ensemble methods modeled complex, nonlinear interactions.

SHAP were computed for each model to quantify the marginal contribution of individual predictors to the probability of PCC uptake. SHAP values were aggregated across individuals to assess global feature importance and to estimate the proportion of total explained variance attributable to effort versus circumstance domains.

Logistic regression was implemented as a baseline model because of its simplicity and interpretability. As logistic regression is generally less sensitive to hyperparameter settings than more complex nonlinear algorithms, the default parameter configuration was used, including L2 regularization with C = 1.0, without further tuning. Hyperparameter tuning for RandomForest and XGBoost was conducted using Bayesian optimization with 5-fold stratified cross-validation, maximizing AUC. Final model parameters and best cross-validation score (AUC) are provided in Supplementary Table 3.

Subgroup analyses explored subgroup effects (education, census registration, annual PCC promotion at local MCH institutions, pregnancy intention). To assess the robustness of the main findings, two sensitivity analyses were performed. First, educational attainment, considered the variable most likely to be subject to classification ambiguity in the Chinese context, was alternatively classified as an effort-related variable to examine the potential impact of misclassification between circumstance- and effort-related variables. Second, a complete-case analysis was conducted to evaluate the influence of missing-data handling on the results. Missing data were handled using multiple imputation by chained equations (MICE).

Python (version 3.9) was employed for models developed and SHAP estimation. R (version 4.3.1) were employed for data imputation, data visualization and other statistical analyses.

## Results

### Participant characteristics and disparities in PCC utilization

Table 1 summarizes the demographic, socioeconomic, and healthcare access characteristics of the 8,866 pregnant women included in the analysis. Most participants were under 35 years old and nulliparous. Despite national provision of free PCC, only 42.4% reported having utilized such services prior to their current pregnancy, with substantial disparities across socioeconomic, geographic, and informational factors. Higher income, higher educational attainment, urban residence, and living in economically developed regions were associated with greater utilization. Access indicators, such as proximity to MCH institutions, participation in annual PCC promotion, and regular physical examinations. Effort-related factors including knowledge and attitude toward PCC, pregnancy intention, parity, and reproductive health history, also corresponded to differences in uptake.

**Table 1:** Characteristics of study participants and associated factors of PCC.

Variable	Total (%)a	Utilized (%)b	Unadjusted			Adjusted		
			Marginal eff (95%CI)	OR (95%CI)	*P* value	Marginal eff (95%CI)	OR (95%CI)	*P* value
Circumstance								
Income (RMB)								
No income	1, 293 (14.6)	441 (34.1)	Ref	Ref	Ref	Ref	Ref	Ref
Less than 5, 000	3, 804 (42.9)	1, 417 (37.3)	0.1671 (0.1314, 0.2028)	1.15 (1.01, 1.31)	0.043	−0.014 (−0.0461, 0.0180)	0.94 (0.83, 1.08)	0.392
5, 001–10, 000	2, 181 (24.6)	1, 094 (50.2)	0.0314 (0.0014, 0.0615)	1.94 (1.69, 2.24)	<0.001	0.0483 (0.0082, 0.0883)	1.22 (1.03, 1.44)	0.018
10, 001 and above	1, 588 (17.9)	807 (50.8)	0.1605 (0.1273, 0.1938)	2.00 (1.72, 2.32)	<0.001	0.0401 (−0.0062, 0.0865)	1.18 (0.97, 1.43)	0.090
Educational attainment								
Elementary school	712 (8.0)	201 (28.2)	Ref	Ref	Ref	Ref	Ref	Ref
Secondary school	2, 747 (31.0)	869 (31.6)	0.0340 (−0.0033, 0.0714)	1.18 (0.98, 1.41)	0.08	0.0316 (−0.0102, 0.0734)	1.14 (0.96, 1.36)	0.138
College/university (bachelor’s degree)	4, 324 (48.8)	2094 (48.4)	0.2020 (0.1657, 0.2382)	2.39 (2.01, 2.85)	<0.001	0.1820 (0.1406, 0.2233)	2.14 (1.80, 2.55)	<0.001
College/University (postgraduate degree)	1, 083 (12.2)	595 (54.9)	0.2671 (0.2227, 0.3115)	3.10 (2.54, 3.80)	<0.001	0.2370 (0.1846, 0.2895)	2.69 (2.16, 3.37)	<0.001
Census registration (urban–rural)								
Rural	5, 705 (64.3)	2, 165 (37.9)	Ref	Ref	Ref	Ref	Ref	Ref
Urban	3, 161 (35.7)	1, 594 (50.4)	0.1248 (0.1033, 0.1463)	1.66 (1.52, 1.82)	<0.001	Not adjusted	Not adjusted	Not adjusted
Region (by economic development level)^c^								
Economically undeveloped regions	5, 719 (64.5)	2, 222 (38.9)	Ref	Ref	Ref	Ref	Ref	Ref
Economically developed regions	3, 147 (35.5)	1, 537 (48.8)	0.0999 (0.0783, 0.1214)	1.50 (1.38, 1.64)	<0.001	Not adjusted	Not adjusted	Not adjusted
Distance to nearest MCH institution (km)								
<5	2, 598 (29.3)	1, 174 (45.1)	Ref	Ref	Ref	Ref	Ref	Ref
5–20	4, 653 (52.5)	2049 (44.0)	−0.0115 (−0.0354, 0.0123)	0.95 (0.867, 1.05)	0.344	−0.0109 (−0.0344, 0.0126)	0.96 (0.87, 1.05)	0.363
>20	1, 615 (18.2)	536 (33.3)	−0.1200 (−0.1499, -0.0901)	0.60 (0.53, 0.69)	<0.001	−0.1030 (−0.1341, -0.0720)	0.65 (0.57, 0.74)	<0.001
Annual PCC promotion at local MCH institutions								
Unaccepted	3, 512 (39.6)	910 (25.9)	Ref	Ref	Ref	Ref	Ref	Ref
Accepted	5, 354 (60.4)	2, 849 (53.2)	0.2730 (0.2533, 0.2927)	3.25 (2.97, 3.57)	<0.001	0.2619 (0.2436, 0.2803)	3.16 (2.88, 3.47)	<0.001
Annual physical examination								
No	4, 195 (47.3)	1, 211 (28.9)	Ref	Ref	Ref	Ref	Ref	Ref
Yes	4, 671 (52.7)	2, 548 (54.5)	0.2568 (0.2370, 0.2766)	2.96 (2.71, 3.23)	<0.001	0.2140 (0.1917, 0.2364)	2.48 (2.24, 2.75)	<0.001
Age (years old)								
≤35	7, 697 (86.8)	3, 026 (39.3)	Ref	Ref	Ref	Ref	Ref	Ref
>35	1, 169 (13.2)	733 (62.7)	0.0424 (0.0154, 0.0693)	1.19 (1.07, 1.32)	0.002	Not adjusted	Not adjusted	Not adjusted
Parity (times)								
0	4, 594 (51.8)	2, 278 (49.6)	Ref	Ref	Ref	Ref	Ref	Ref
1	2, 916 (32.9)	1, 070 (36.7)	−0.1289 (−0.1516, -0.1062)	0.59 (0.53, 0.65)	<0.001	−0.0875 (−0.1110, -0.0640)	0.70 (0.63, 0.77)	<0.001
2	1, 356 (15.3)	411 (30.3)	−0.1928 (−0.2212, -0.1644)	0.44 (0.39, 0.50)	<0.001	−0.1005 (−0.1336, -0.0674)	0.66 (0.58, 0.76)	<0.001
Knowledge of PCC								
Knowledge of PCC services								
Poor	2, 406 (27.1)	157 (6.5)	Ref	Ref	Ref	Ref	Ref	Ref
Good	2, 637 (29.7)	944 (35.8)	0.2927 (0.2719, 0.3135)	7.99 (6.69, 9.60)	<0.001	0.2153 (0.1836, 0.2471)	2.73 (2.34, 3.18)	<0.001
Excellent	3, 823 (43.1)	2, 658 (69.5)	0.6300 (0.6124, 0.6476)	32.68 (27.50, 39.10)	<0.001	0.4888 (0.4609, 0.5166)	9.75 (8.34, 11.43)	<0.001
Knowledge of PCC policy								
No	4, 746 (53.5)	1, 299 (27.4)	Ref	Ref	Ref	Ref	Ref	Ref
Yes	4, 120 (46.5)	2, 460 (59.7)	0.3234 (0.3038 0.3430)	3.93 (3.60, 4.30)	<0.001	0.1807 (0.1591, 0.2023)	2.14 (1.94, 2.36)	<0.001
Health status								
Common infectious diseases, chronic diseases and other diseases								
No	8, 174 (92.2)	3, 446 (42.2)	Ref	Ref	Ref	Ref	Ref	Ref
Yes	692 (7.8)	313 (45.2)	0.0307 (−0.0079, 0.0693)	1.13 (0.97, 1.32)	0.116	0.0199 (−0.0183, 0.0581)	1.08 (0.931.27)	0.308
Adverse pregnancy outcomes								
No	6, 077 (68.5)	2, 496 (41.7)	Ref	Ref	Ref	Ref	Ref	Ref
Yes	2, 789 (31.5)	1, 263 (45.3)	0.0421 (0.0199, 0.0644)	1.19 (1.08, 1.30)	<0.001	0.0420 (0.0198, 0.0643)	1.19 (1.08, 1.30)	<0.001
Reproductive system diseases								
No	6, 645 (74.9)	2, 684 (40.4)	Ref	Ref	Ref	Ref	Ref	Ref
Yes	1, 308 (14.8)	689 (52.3)	0.1228 (0.0933, 0.1524)	1.64 (1.46, 1.85)	<0.001	0.1435 (0.1167, 0.1704)	1.81 (1.62, 2.03)	<0.001
Not clear	913 (10.3)	386 (42.3)	0.0189 (−0.0153, 0.0530)	1.08 (0.94, 1.24)	0.276	0.0351 (0.0013, 0.0688)	1.16 (1.01, 1.33)	0.0418
Pregnancy intention								
Unplanned	3, 751 (42.3)	696 (18.6)	Ref	Ref	Ref	Ref	Ref	Ref
Planned	5, 115 (57.7)	3, 063 (59.9)	0.4133 (0.3950, 0.4316)	6.55(5.93, 7.24)	<0.001	0.3698 (0.3562, 0.3833)	6.13 (5.56, 6.76)	<0.001
Attitude toward PCC								
Negative	2, 111 (23.8)	394 (18.7)	Ref	Ref	Ref	Ref	Ref	Ref
Positive	6, 755 (76.2)	3, 365 (49.8)	0.3115 (0.2911, 0.3320)	4.33 (3.84, 4.88)	<0.001	0.1979 (0.1727, 0.2230)	2.30 (2.06, 2.57)	<0.001

^a^Number of participants (Percentage of participants).

^b^Number of participants (Utilized rate).

^c^Cities with a per capita GDP over 120,000 RMB: economically developed; cities with a per capital GDP below 120,000 RMB: economically underdeveloped.

Not adjusted, these variables not adjusted confounding variables.

PCC, preconception care; MCH, maternal and child health institution.

Variables were residualized based on a directed acyclic graph (DAG) and Spearman correlation analysis. Adjusted estimates were then obtained using logistic regression models based on the residualized variables. Variables that were not residualized were not included in the adjusted analyses in this study.

### Associated factors of PCC utilization

Adjusted analyses using the EOp framework and a DAG-informed causal structure revealed that both structural and individual factors independently influenced PCC uptake. Socioeconomic factors, including income and education, remained strong predictors, as did urban residence and proximity to care. Informational and access-related variables, such as participation in annual PCC promotion and physical examinations, independently increased utilization. Among effort-related predictors, knowledge of PCC services, pregnancy intention, and positive attitude toward PCC showed the largest effects. Higher parity was negatively associated, whereas older maternal age, adverse pregnancy outcomes, and reproductive system diseases were positively associated.

### Machine learning model performance and SHAP-based decomposition

To quantify predictors’ contributions to disparities in PCC utilization and evaluate their robustness, we developed three classifier models, logistic classifier, RandomForest, and XGBoost, using the same set of DAG-informed predictors. To provide additional context for the fitted models underlying the SHAP analysis, model performance metrics are reported in Supplementary Table 4. Overall, all models demonstrated satisfactory performance and discriminative ability. Their results across multiple evaluation metrics also suggested a good balance between correctly identifying positive and negative cases. Taken together, these findings indicate that the models were well fitted and robust, thereby strengthening the credibility of the subsequent SHAP-based interpretation of variable importance. As no independent external validation was performed, these results should be interpreted within the context of the present dataset and modeling framework.

**Figure 2:**
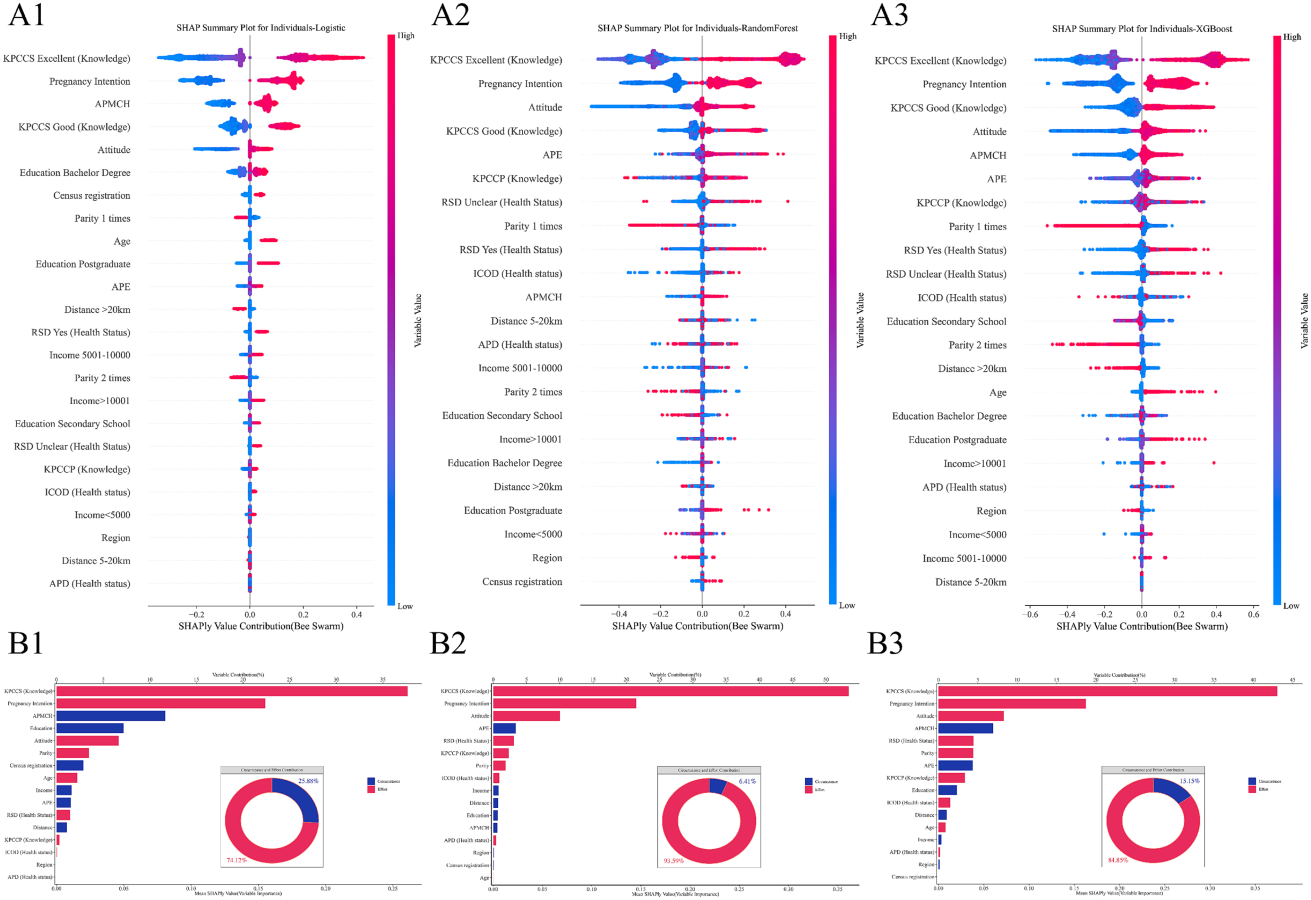
Interpretation of models and contribution of variables to PCC utilization based on SHAP decomposition. **(A)** Models’ interpretation. **(B)** Variable contributions identified by SHAP value decomposition. Models’ interpretation: variables are ranked by their mean absolute SHAP values, with the position of each point determined by the SHAP value and the color gradient indicating the distribution of original feature values. Distance: distance to nearest maternal and child health institution, AMPCH, annual PCC promotion at local maternal and child health (MCH) institutions; APE, annual physical examination, KPCCS, knowledge of PCC services, KPCCP, knowledge of PCC policy; ICOD, common infectious diseases, chronic diseases, and other diseases; APD, adverse pregnancy outcomes; RSD, reproductive system diseases; Attitude, attitude toward PCC.

Figure 2 presents the SHAP summary plots for each model and summarizes the aggregate contribution of circumstance and effort domains. In the Figure 2A, variables are ranked by their mean absolute SHAP values, with the position of each point determined by the SHAP value and the color gradient indicating the distribution of original feature values. The detailed SHAP values and contributions of each variable are displayed in Figure 2B. Across all three models, the most influential predictors were consistently effort-based factors: knowledge of PCC services and pregnancy intention. Notably, effort-related variables explained the majority of predicted variation, contributing 74.1, 93.6, and 84.9% of total model importance in logistic regression, RandomForest, and XGBoost, respectively. Among circumstance factors, annual physical examination emerged as the leading predictor in the RandomForest model, whereas annual PCC promotion at local MCH institutions was the most influential in the logistic classifier and XGBoost models. These structural predictors played a secondary but non-negligible role, highlighting the interplay between socioeconomic context and personal agency. Overall, the SHAP decomposition findings validate and expand upon the regression-based results. Across models, knowledge-related variables and pregnancy planning consistently ranked among the most important factors associated with PCC utilization.

### Subgroup and sensitivity analyses

**Figure 3:**
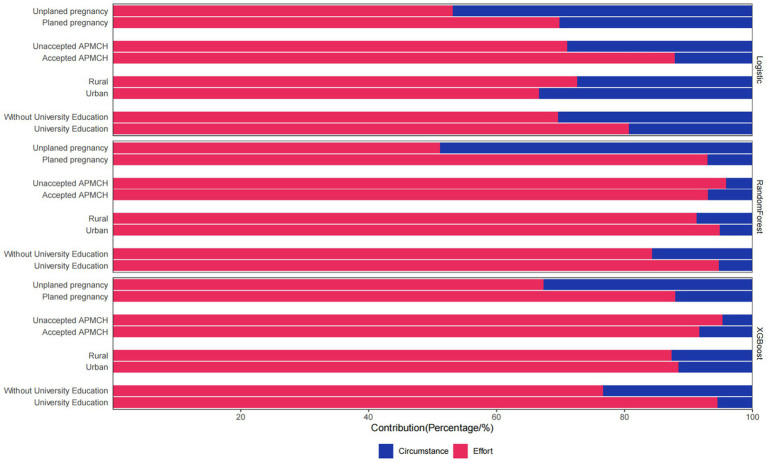
Subgroup analysis of effort and circumstance contributions to PCC utilization. AMPCH, annual PCC promotion at local maternal and child health (MCH) institutions.

We observed meaningful subgroup differences in the relative contributions of effort and circumstance variables across education level, urban–rural residents, whether or not the annual PCC promotion was accepted at local MCH institutions, and pregnancy intention status, as shown in Figure 3. The detailed contributions of the variables are provided in Supplementary Tables 5–7. Among participants without a university education, circumstance variables explained a substantially larger share of PCC utilization inequality, accounting for 15.7 to 30.4% of predictive importance, compared to just 5.3 to 19.3% among those with higher education. Differences by urban–rural residence were modest across models. In RandomForest and XGBoost, rural residents were more influenced by effort-related factors than urban residents. However, the opposite result emerged in logistic regression. Among women who accepted PCC promotion, circumstance variables contributed more to inequality in the machine learning models. Conversely, in the logistic regression model, women unaccepted PCC promotion exposure showed higher circumstance contributions (29.0%), indicating model-dependent variation. The most striking pattern emerged in relation to pregnancy intention. For women with planned pregnancies, effort variables dominated (69.8–93.0% across models). In contrast, women with unplanned pregnancies showed higher circumstance contributions, especially in logistic classifier and RandomForest model, explaining nearly half of variation across models.

The sensitivity analyses demonstrated that the main findings were robust. Reclassifying educational attainment as an effort-related variable produced contribution patterns broadly consistent with those of the main analysis. Similarly, the complete-case analysis yielded comparable results (Supplementary Figure 2), indicating that the overall findings were not materially affected by either the alternative specification of education or the handling of missing data.

## Discussion

### Summary of key findings

This study provides one of the first empirical applications of the EOp framework to examine PCC utilization in China, using nationally distributed data and advanced machine learning explainability tools. Our findings reveal that despite free, universal PCC provision, overall uptake remains suboptimal and socially stratified. SHAP-based decomposition indicated that effort variables dominate overall, but structural constraints remain decisive, especially in disadvantaged subgroups.

### Interpretation and mechanism

Our analyses indicate that individual “effort” factors—particularly knowledge of PCC services and pregnancy planning—are the strongest determinants of utilization. The absence of knowledge of PCC among women had substantially lower uptake rates, highlighting a pervasive knowledge gap that remains unaddressed by current outreach efforts. This aligns with previous research emphasizing the central role of awareness in health-seeking behaviors (20). In addition, when pregnancies are unintended, the opportunity for preconception care is often missed, contributing to lower uptake (21). Globally, unintended pregnancies remain common (22), and evidence from sub-Saharan Africa shows that they are associated with markedly reduced use of preconception services (23, 24). The dominant role of effort variables, particularly pregnancy planning and PCC knowledge, may suggest that increasing individual agency could reduce disparities. However, this interpretation must be qualified. Although the classification of variables into “effort” and “circumstance” was guided by Roemer’s framework, the distinction is not always absolute in empirical research. Some variables may contain both agency-related and context-dependent components. For example, knowledge of PCC was classified as an effort variable in this study because it is more proximal to individual awareness and behavioral readiness. However, such knowledge is also shaped by broader contextual factors, including educational attainment, access to health information, and service outreach. Therefore, this classification should be understood as a theory-informed operational approach rather than a strict ontological separation. Our residualization approach, supported by DAG-informed modeling, attempted to statistically isolate these domains, yet real-world behaviors are inevitably shaped by both.

Notably, our results showed that even factors like health status and parity influenced PCC use. Women with pre-existing medical conditions or advanced age were more likely to seek PCC (perhaps due to heightened risk perception), whereas multiparous women were less likely to do so. This is consistent with studies linking higher parity to lower uptake of preventive health behaviors, possibly because experienced mothers feel more confident or face time constraints, or because health workers focus PCC outreach on first-time parents (25, 26). The tendency for healthier or younger individuals to under-utilize PCC (underestimating their risk) has also been observed in other contexts. These nuances point to the need for tailored messaging. PCC promotion must target not only the classically underserved (rural, low-income, less educated) but also groups who may be overlooked, such as multiparous women and those who appear healthy. Everyone of reproductive age stands to benefit from preconception guidance, not just those with obvious risk factors. Interestingly, pregnant women with infectious or chronic conditions did not exhibit increased utilization of PCC services. This may be due to their greater focus on health management, prompting them to seek more specialized care from other healthcare institutions rather than relying solely on preconception services.

Structural factors also emerge as critical. We found that living in a rural area, low income, and unaccepted PCC promotion at local MCH institutions were all associated with lower PCC use, even though direct financial cost was removed by the free program. This aligns with global evidence that making services available is necessary but not sufficient to achieve equitable uptake (27, 28). Rural women often face shortages of healthcare resources and greater travel distances, while those with lower education may lack awareness of PCC’s benefits. These disadvantages tend to cluster: for example, rural residents in China are more likely to be poor, less educated, and lack access to information, compounding their lower utilization of PCC. Indirect costs, such as transportation or lost wages, also likely deter the poorest from attending even “free” services. Notably, exposure to PCC promotion campaigns was the most influential circumstance variable in our analysis, indicating that information inequity is a major driver of the gap. Inadequate public health outreach can leave entire communities unaware of PCC options, a phenomenon not unique to China. Studies in Africa similarly cite “lack of awareness about the availability of services” as a dominant reason for not utilizing PCC (29). The urban–rural health awareness gap evidenced in our study echoes the experience of many low- and middle-income countries (LMICs), where rural women receive less health information and face greater barriers to care. It is telling that even in prosperous urban centers like Shanghai, only about 42% of couples report attending preconception services, indicating that beyond structural access, a generalized low priority or knowledge of PCC persists in the population (28).

### Comparison with global evidence

Placing the Chinese experience in a global context, we find both resonances with and contrasts to other countries. On one hand, the fundamental barriers to PCC utilization in China, education, income, region, and pregnancy intention, are broadly mirrored in other LMICs. For example, a recent meta-analysis estimated PCC utilization in Ethiopia at only 16%, and in Nigeria it can be as low as 10%, with low education and poor awareness repeatedly identified as limiting factors (20, 30). Across Africa, fewer than one in five pregnant women have received any preconception care (31). In these settings, just as in China, rural young women with minimal schooling are least likely to access pre-pregnancy services.

High-income countries (HICs) likewise grapple with inequities in preconception health, albeit in different forms. Many HICs do not have formal PCC programs like China’s, but they typically have routine primary care or obstetric services where preconception advice can be given (32, 33). Yet, studies show uptake remains suboptimal and socially patterned. In the United Kingdom, about half of pregnancies are unplanned and a staggering 90% of women enter pregnancy with at least one modifiable risk factor (such as smoking, obesity, or lacking folic acid supplementation) (34). These risk factors are more prevalent among disadvantaged groups. Such disparities echo our findings in China that structural disadvantage translates into poorer preconception health preparation. In the United States, despite extensive healthcare resources, similar patterns are observed (35). Unintended pregnancy is also concentrated among the less advantaged in the U.S., with women of color and those lacking health insurance experiencing markedly higher rates of unplanned pregnancies (36). These HIC examples reinforce a key point: structural inequities spare no country. Whether in a low-income rural province in China or an inner-city community in the U.S., social determinants, income, education, race, geography, shape individuals’ opportunities to prepare for a healthy pregnancy (37, 38). The commonalities across such different contexts strengthen the case that addressing these upstream factors is both an ethical imperative and a practical necessity to improve maternal-child health outcomes globally.

The Chinese context may help explain why effort-related factors accounted for a relatively large share of inequality in PCC utilization. Unlike settings where preconception care is more often initiated through individual demand within clinic-based systems, PCC in China is embedded within a publicly funded maternal and child health service framework and is influenced by local outreach, community-level mobilization, and regional service delivery capacity. Even within such a system, however, actual uptake still depends heavily on individual awareness, pregnancy planning, and related behavioral factors. These effort-related factors may also be shaped by broader sociocultural influences, including family involvement in reproductive decision-making, gendered expectations surrounding pregnancy preparation, and differences in health awareness and service utilization across social groups. In this sense, effort-related factors may play a prominent role, although they remain conditioned by broader structural and service-delivery contexts.

### Policy implications

By quantifying the contributions of circumstances vs. effort, our analysis provides insight into where policy levers might be most effective. The finding that effort-related factors explained roughly 74.1–93.6% of the inequality suggests that much of the gap in PCC use could be closed by influencing personal-level factors such as awareness and motivation. Yet, we caution that these personal factors do not operate in a vacuum, increasing someone’s “effort” (e.g., encouraging them to plan pregnancies or seek PCC) will only succeed if the circumstance enables it. In subgroups like women with unplanned pregnancies, we saw circumstance and effort factors nearly equally dominating PCC utilization, indicating that when personal preparedness is low, social conditions become even more determinative. The EOp approach thus reinforces a dual obligation for policymakers: to empower individuals with knowledge, resources, and support, and simultaneously to dismantle structural barriers that constrain certain groups’ ability to utilize care. It also brings a normative clarity, highlighting that the inequalities we observe are not value-neutral, but involve remediable injustices. In line with the principles espoused by the WHO and the Lancet Commission on health inequities (38), we affirm that differences in PCC uptake linked to circumstances like rural poverty or low education are ethically unacceptable and merit focused redress.

Policy implications from this study are both domestic and global. For China, the results call for a recalibration of strategies to ensure the next mile of PCC coverage reaches those left behind. Despite the success of the national PCC program in scaling up services, our data suggest that marginalized subpopulations (rural, less educated, low-income, unplanned pregnancies) continue to have lower utilization. Bridging this gap requires an equity-focused redesign of interventions.

Existing PCC models in China—including the “Three-in-One,” “Three-Combination,” “City-District Integrated,” “County-Town Joint Service”, “Combined Premarital and Preconception Examination”, and “Guangxi Comprehensive Service”—have used grid-based management, interdepartmental coordination, one-stop services, mobile clinics, and digital platforms to improve awareness and uptake. However, important implementation challenges remain, including variation in local implementation capacity, limited integration between primary healthcare and maternal and child health services, uneven outreach to underserved women, and persistent geographic and social disparities in access. From a policy perspective, improving PCC utilization in China may require more than broad public promotion alone. First, PCC could be strengthened through primary healthcare institutions, such as community health service centers and township health centers, by incorporating routine counseling, risk screening, and referral into existing reproductive and maternal health services. Second, targeted outreach efforts may be needed for women in rural or socioeconomically disadvantaged settings, who may face greater barriers to information and access. Strengthening coordination between primary care providers and maternal and child health institutions may help create a more continuous and accessible PCC service pathway.

Internationally, the implications extend to all countries striving to meet the Sustainable Development Goals (SDG) targets for maternal and child health (39). Global health actors should recognize PCC as a critical but underutilized lever for improving maternal-newborn outcomes and reducing inequities. Bodies like the WHO, UNFPA, and the Lancet commissions have increasingly pointed out that progress in maternal health has stalled and that new approaches are needed (40). Our study’s insights support a reorientation of maternal health policies to include robust preconception strategies. Governments and donors must invest in programs that embed equity into PCC rollout, ensuring that the benefits of preconception interventions accrue to all segments of society, not just the privileged.

### Strengths and limitations

This study presents several notable contributions to advancing equity in maternal health. First, it is among the few to apply EOp framework to PCC, offering a normative lens to distinguish ethically actionable disparities from those linked to personal agency. This conceptual grounding enhances the policy relevance of our findings. Second, by combining SHAP-based decomposition with interpretable machine learning models (logistic regression, RandomForest, XGBoost), we provide a robust method for quantifying the relative contributions of effort and circumstance variables to inequality. The inclusion of subgroup-specific SHAP analyses enables deeper insight into differential drivers of PCC uptake across populations. Third, we employed residualization and DAG-informed modeling to minimize attribution bias between effort and circumstance factors. This strengthens the analytical alignment with the EOp framework and enhances interpretability. Importantly, this study draws on data from one of the world’s largest publicly funded PCC programs, offering transferable insights for other low- and middle-income countries (LMICs) seeking to reduce reproductive health inequalities. The finding that substantial disparities persist despite universal service availability underscores the limits of supply-side expansion alone.

Nonetheless, this study has several limitations. First, the cross-sectional design precludes causal inference. Second, healthcare facilities were selected purposely rather than through probability-based sampling. Although this strategy was used to increase contextual heterogeneity, it may have introduced selection bias, as facilities with greater willingness or stronger operational capacity may have been more likely to participate. Therefore, the findings should be generalized with caution. Third, PCC utilization was self-reported and may therefore be subject to recall bias or social desirability bias, potentially leading to misestimation of the true uptake rate. In addition, some variables may not fit neatly into a single category of “effort” or “circumstance.” This potential overlap may have introduced a degree of misclassification, which could influence the estimated relative contributions of the two domains. Accordingly, the decomposition results should be interpreted with appropriate caution. Despite these limitations, this study offers a rigorous, equity-focused analysis of PCC uptake. By integrating theory, advanced analytics, and policy relevance, it provides practical guidance for designing more just and inclusive reproductive health systems.

## Conclusion

In conclusion, inequalities in PCC utilization persist despite the availability of a universal, publicly funded PCC system. Using the Equality of Opportunity framework and SHAP-based decomposition, this study offers an equity-oriented perspective on the factors underlying these disparities. Future research should adopt longitudinal designs to clarify temporal relationships and better assess how inequities in PCC utilization develop over time. Such evidence will be important for informing more equitable maternal health policies and interventions.

## Data Availability

The raw data supporting the conclusions of this article will be made available by the authors, without undue reservation.
