# Supplementary material for: Inequality in preconception care utilization based on the opportunity inequality framework: evidence from explained machine learning
Source: Front Public Health. 2026 May 7;14:1732192. doi: 10.3389/fpubh.2026.1732192 (PMC13190449; doi:10.3389/fpubh.2026.1732192)
Supplement: Supplementary file 1 [file Data_Sheet_1.DOCX]

**Supplementary Text 1**

To address dependency among explanatory variables, a residualization procedure was implemented based on the combined results of the correlation analysis and the Directed Acyclic Graph (DAG)-informed causal structure. The purpose of this approach was to isolate the variation in each predictor that remained after accounting for its dependency on upstream variables, thereby reducing redundancy among predictors and improving interpretability in subsequent analyses. For each variable considered to be influenced by earlier variables in the DAG, a regression model was fitted using its presumed parent variables as predictors, and the residualized value was defined as the component not explained by those upstream predictors. For binary variables, logistic regression was applied, and the residual was calculated as the observed value (coded as 0/1) minus the model-predicted probability. For multinomial variables, the residualization procedure was based on the corresponding category-specific predicted probabilities.

Residualization procedure and data transformer:

1. We referred to relevant literature to establish causal relationships between explanatory variables and constructed a Directed Acyclic Graph (DAG) diagram to determine causal directions.
2. We built Logistic regression models among the explanatory variables.
3. Using the models we developed, we predicted the outcomes of the model.

P(y_i_ = 1|x_i1_, x_i2_,…,x_ij_) = $\frac{1}{1 + e^{-(\beta_{0}+\beta_{1}X_{1}+\beta_{2}X_{2}+\ldots+\beta_{j}X_{j})}}$

P(y_i_ = k|x_i1_, x_i2_,…,x_ij_) = $\frac{e^{\beta_{k0}+\beta_{k1}X_{i1}+\beta_{k2}X_{i2}+\ldots+\beta_{\mathrm{kj}}X_{\mathrm{ij}}}}{\sum_{c=1}^{K}e^{\beta_{c0}+\beta_{c1}X_{i2}+\ldots+\beta_{cj}X_{ij}}}$

β: Represents the coefficients of the model

xij : Represents the jth variable or variable of the ith individual

k: Represents a specific class or category, typically ranging from 1 to K

K: Represents the total number of classes or categories in a classification problem

P(y_i_ = 1|x_i1_, x_i2_,…,x_ij_) represents the probability that the target variable y_i_ equals 1 given the features x_i1_, x_i2_,…,x_ij_ of the ith individual

P(y_i_ = k|x_i1_, x_i2_,…,x_ij_): Represents the probability that the ith individual belongs to class k given the feature values

1. The residual values of the explanatory variables were calculated as the observed values of the variables minus the predicted probabilities of the variables.

E_i_ = Actually Probability - P(x_ij_ = 1|x_i1_, x_i2_,…,x_ij_)

E_i_ = Actually Probability - P(x_ij_ = k|x_i1_, x_i2_,…,x_ij_)

Ei: Represents the residual of the ith individual

**Supplementary Figure 1: Provinces surveyed and distribution in this study**


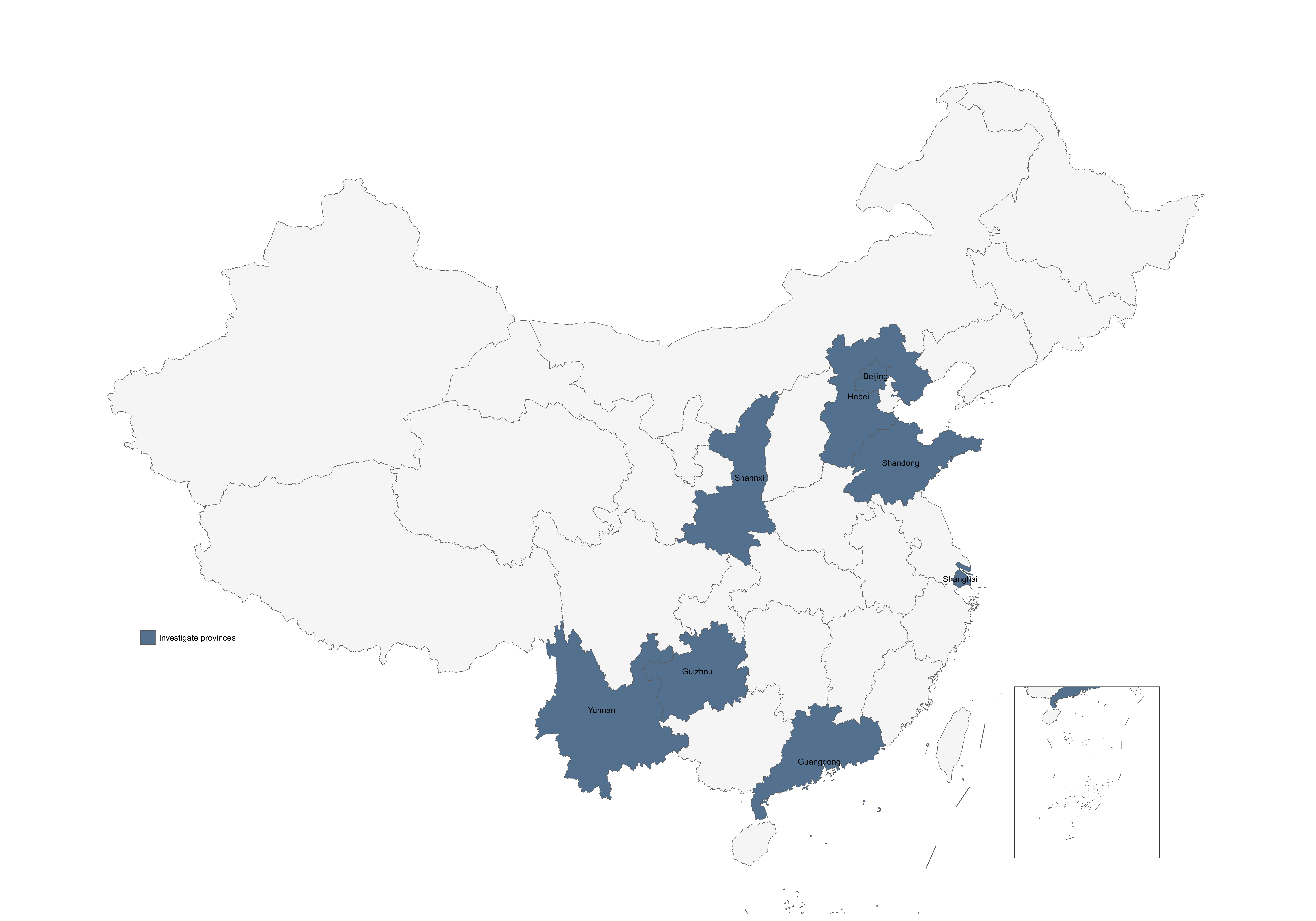


**Supplementary Figure 2: Sensitivity analysis**


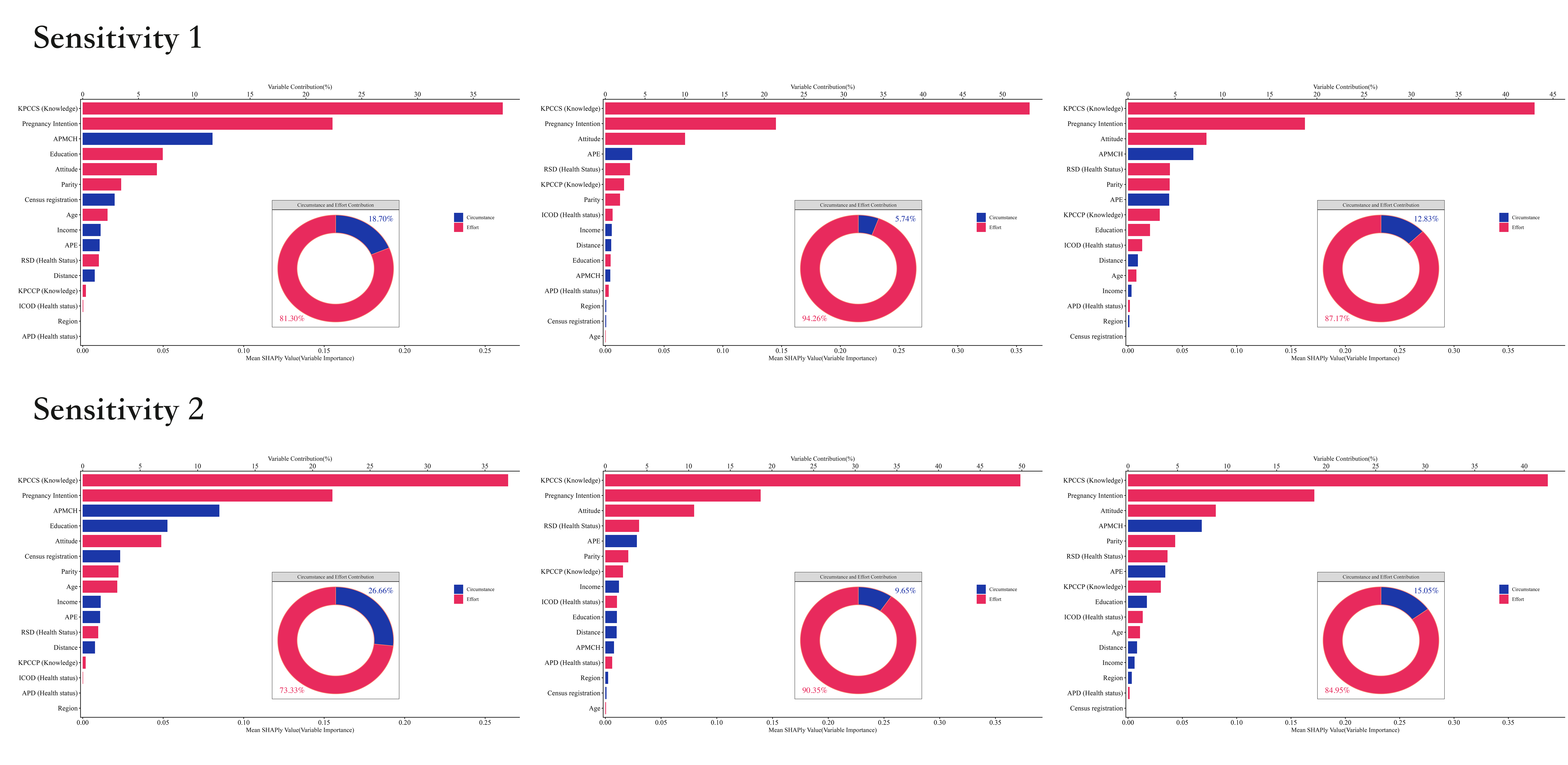


**Supplementary Table 1: Study variables**

| Variables | Original questions in the questionnaire | Type of variables | Supplementary explanation |
| --- | --- | --- | --- |
| PCC | Have you ever accepted preconception care?  1.No 2.Yes  Supplementary question:   1. Have you undergone preconception risk assessment, including preconception counseling and training? 2. Have you received preconception health guidance, including the guidance for lifestyle and nutrition for preconception? 3. Have you had preconception health examination, such as blood tests and gynecological examinations? | Outcome | The utilization of PCC is primarily obtained through inquiry. When the participants have received any of the services in the supplementary question mentioned, it is also considered as having received PCC. |
| Income | Your monthly income after tax before pregnancy is?  1.No income 2.<5000RMB 3.5001-10000RMB 4>10001RMB | Circumstance | Typical socioeconomic variables |
| Educational Attainment | What is your level of education?  1.Elementary School 2.Secondary School  3.College/University (Bachelor's Degree)  4.College/University (Postgraduate Degree) | Circumstance | Typical socioeconomic variables |
| Census Registration | What is your current household census registration type?  1.Rural 2.Urban | Circumstance | The structural disparities, socioeconomic gaps, and policy variations between urban and rural areas primarily reflect the influence of external circumstance and institutional factors on healthcare service utilization, rather than individual efforts. |
| Region | This variable was obtained through the source of the questionnaire. Since our study subjects are required to have resided locally for more than 6 months, this variable is obtained from the questionnaire source and is considered to be reliable and trustworthy.   1. Economically Developed Regions (Cities with a per capital GDP over 120,000 RMB) 2. Economically Underdeveloped Regions (Cities with a per capital GDP below 120,000 RMB) | Circumstance | Region including geographical location, population density, economic development level, among other characteristics, have a significant impact on the accessibility and utilization of healthcare services. In our study, the region is considered as a circumstance variables rather than a result of individual efforts. |
| Distance to Nearest Maternal and Child Health Institution | How far is the nearest healthcare facility providing preconception care services from your location?   1. Short distance (<5km) 2. Medium distance (5-20km) 3. Long distance (>20km) | Circumstance | Distance can impact the convenience of individuals accessing healthcare services, including factors such as the accessibility of healthcare facilities and the ease of transportation. In our study, the region is considered as a circumstance variables rather than a result of individual efforts. |
| Annual PCC Promotion at Local MCH Institutions | Have you attended any community or hospital promotional activities related to preconception health or health promotion?  1.No 2.Yes | Circumstance | The successful implementation of promotional activities for preconception health services and health promotion is largely influenced by societal support, policy support, and resource allocation. Therefore, it is considered a circumstance variable. |
| Annual Physical Examination | Do you undergo a medical check-up at least once a year?  1.No 2.Yes | Circumstance | In China, physical examination not free, and the frequency of undergoing at least one physical examination per year is largely influenced by factors such as occupation, socioeconomic status, healthcare accessibility, among others. In this study, it is considered to be more of a circumstance variable. |
| Age | How old are you this year? | Effort | Age, as a physiological indicator, reflects the research subject's demand for healthcare services. |
| Parity | How many times have you given birth?  1.zero 2.one 3.three and over | Effort | The parity (number of pregnancies) is first and foremost an individual's choice. |
| Knowledge of PCC Services | Did you know about PCC before pregnancy?   1. No 2.Yes   Did you know what services items are included in PCC?   1. No 2.Yes   Participants do not know about PCC were defined poor knowledge.  Participants know about PCC were defined good knowledge.  Participants know about PCC services items were defined excellent knowledge. | Effort | Reflect on the knowledge of PCC. Self-acquiring knowledge in this area is a reflection of personal choice and effort, as well as the influence of personal willingness, motivation, and self-efficacy on behavior. |
| Knowledge of PCC Policy | Did you know that preconception health care is free before pregnancy?  1.No 2.Yes | Effort | Reflect on the knowledge of PCC. Self-acquiring knowledge in this area is a reflection of personal choice and effort, as well as the influence of personal willingness, motivation, and self-efficacy on behavior. |
| Common Infectious Diseases, Chronic Diseases and Other Diseases | Have you ever had any chronic diseases or infectious diseases before pregnancy?  1.No 2.Yes  Including following diseases  1.Hypertension 2.Diabetes 3.Heart disease 4.Tuberculosis  5.Anemia 6.Hepatitis 7.Nephritis 8.Rheumatism  9.Blood disorder 10.History of allergies 11.Depression  12.Other mental illnesses 13.Genetic diseases | Effort | Reflect on the health status. Reflect on healthcare demand. |
| Adverse Pregnancy  outcome | Have you had any of the following adverse pregnancy outcomes before pregnancy?  1.No 2.Yes  Including following diseases  1. Miscarriage 2.Ectopic pregnancy 3.Premature birth  4.Stillbirth 5.Gestational diabetes 6.Gestational hypertension  7.Pregnancy infection  8.Neonatal diseases (macrosomia, jaundice, hypoglycemia, birth asphyxia, etc.)  9.Birth defects | Effort | Reflect on the health status. Reflect on healthcare demand. |
| Reproductive Diseases | Did you have any reproductive system diseases before pregnancy, such as vaginitis, cervicitis, salpingitis, etc.?  1.No 2.Yes 3.Not clear | Effort | Reflect on the health status. Reflect on healthcare demand. |
| Pregnancy Intention | Was this pregnancy planned? | Effort | Personal decision |
| Attitude towards PCC | Your attitude towards preconception care？  1.Completely disregarding 2.Little disregarding 3.Generally  4.Important 5.Very important  Completely disregarding, Little disregarding, Generally were defined as negative attitude.  Important and Very important were defined as Positive attitude. | Effort | Personal willingness |

**Supplementary table 2: Decision of possible causal connections in DAG**

| Variables (Outcomes) | Possible causal connections in DAG (Exposure) |
| --- | --- |
| Income | Age^1–3^, Census Registration^4–8^,  Regions^9,10^, Educational Attainment^11–14^ |
| Educational Attainment | Regions^2,15,16^, Census registration^16–18^ |
| Census Registration (Urban-Rural) | Not adjusted |
| Regions | Not adjusted |
| Distance to Nearest MCH | Census registration^19–21^ |
| Annual PCC Promotion at Local MCH Institutions | Distance to Nearest MCH^22^ |
| Annual physical examination | Income^22–24^, Educational Attainment^22–25^,  Census Registration^22,26^, Regions^22^,  Health Status^22–25^, Age^22,23,25^ |
| Age | Not adjusted |
| Parity | Income^27–29^, Educational Attainment^29,30^ |
| Knowledge of PCC | Income^22^, Educational Attainment^31–33^,  Census Registration^34,35^, Regions^34,36^,  Annual PCC Promotion at Local MCH Institutions^31^,  Parity^31,32^, pregnancy intention^33,37^ |
| *Knowledge of PCC Services* |  |
| *Knowledge of PCC Policy* |  |
| Health Status | Income^38–41^, Educational Attainment^42,43^,  Census Registration^35,44^, Age^45^ |
| *Common infectious diseases, chronic diseases and other disease* |  |
| *Adverse pregnancy outcomes* |  |
| *Reproductive system diseases* |  |
| Pregnancy Intention | Income^46–48^, Educational Attainment^49,50^,  Census Registration^50,51^, Regions^52^ |
| Attitude towards PCC | Income^53^, Educational Attainment^54,54^,  Census Registration^55^, Regions^56^,  Annual PCC Promotion at Local MCH Institutions^54^ |

Reference

1. Ozhamaratli F, Kitov O, Barucca P. A generative model for age and income distribution. EPJ Data Sci. SpringerOpen; 2022 Dec;11(1):1–26.

2. PK. Income Percentile by Age Calculator - DQYDJ [Internet]. DQYDJ – Don’t Quit Your Day Job... [cited 2025 Jun 5]. Available from: https://dqydj.com/income-percentile-by-age-calculator/

3. Zajacova A, Lawrence EM. The Relationship Between Education and Health: Reducing Disparities Through a Contextual Approach. Annual Review of Public Health. 2018 Apr 1;39(1):273–289.

4. Wang M, Li B. Urban-rural income gap and urban crime rate. Finance Research Letters. 2024 May 1;63:105285.

5. Sicular T, Ximing Y, Gustafsson B, Shi L. THE URBAN–RURAL INCOME GAP AND INEQUALITY IN CHINA. Review of Income and Wealth. 2007;53(1):93–126.

6. Sicular T, Ximing Y, Gustafsson B, Li S. The Urban-Rural Income Gap and Income Inequality in China. In: Wan G, editor. Understanding Inequality and Poverty in China [Internet]. London: Palgrave Macmillan UK; 2008 [cited 2025 Jun 5]. p. 30–71. Available from: http://link.springer.com/10.1057/9780230584259_2

7. Zhao X, Hao G, Wang Y, Xie X, Wen X, Cao L, Ye S, Lin M. Analysis of the Urban-Rural Income Gap in China. Journal of Sociology and Ethnology. Clausius Scientific Press; 2022 Aug 4;4(6):46–56.

8. Yan D, Sun W, Li P, Liu C, Li Y. Effects of economic growth target on the urban–rural income gap in China: An empirical study based on the urban bias theory. Cities. 2025 Jan 1;156:105518.

9. He S ling, Zhong Y, He W wei. The impact of city size on income inclusive growth: A human capital perspective and evidence from China. PLOS ONE. Public Library of Science; 2024 Feb 12;19(2):e0288294.

10. Zhao S. Disposable income in major cities climbs despite pandemic [Internet]. [cited 2025 Jun 4]. Available from: https://global.chinadaily.com.cn/a/202103/22/WS6058455aa31024ad0bab0c09.html

11. THE IMPACT OF EDUCATION ON INCOME DISTRIBUTION - Tinbergen - 1972 - Review of Income and Wealth - Wiley Online Library [Internet]. [cited 2025 Jun 4]. Available from: https://onlinelibrary.wiley.com/doi/abs/10.1111/j.1475-4991.1972.tb00865.x

12. Fields GS. Education and Income Distribution in Developing Countries: A Review of the Literature. 1980 Jul 1 [cited 2025 Jun 4]; Available from: https://hdl.handle.net/1813/75505

13. DOES EDUCATION REDUCE INCOME INEQUALITY? A META‐REGRESSION ANALYSIS - Abdullah - 2015 - Journal of Economic Surveys - Wiley Online Library [Internet]. [cited 2025 Jun 4]. Available from: https://onlinelibrary.wiley.com/doi/abs/10.1111/joes.12056

14. Education and Economic Rewards. Variations by Social‐Class Origin and Income Measures | European Sociological Review | Oxford Academic [Internet]. [cited 2025 Jun 4]. Available from: https://academic.oup.com/esr/article/17/3/209/545644?login=true

15. Hannum E, Wang M. Geography and educational inequality in China. China Economic Review. 2006 Jan 1;17(3):253–265.

16. Zhao XB, Tong SP. Unequal Economic Development in China: Spatial Disparities and Regional Policy Reconsideration, 1985-1995. Regional Studies. 2000 Aug;34(6):549–561.

17. Guo Y, Li X. Regional inequality in China’s educational development: An urban-rural comparison. Heliyon. 2024 Feb 29;10(4):e26249. PMCID: PMC10877417

18. Interrogating institutionalized establishments: urban–rural inequalities in China’s higher education | Asia Pacific Education Review [Internet]. [cited 2025 Jun 4]. Available from: https://link.springer.com/article/10.1007/s12564-013-9262-0

19. Zhong H, Xiong H, Zhou Y, Liao Y, Wan Y, Lei C. [Comparative Analysis of Influencing Factors of Health Service Utilization Among Urban and Rural Residents in Tibet]. Sichuan Da Xue Xue Bao Yi Xue Ban. 2023 Sep;54(5):985–993. PMCID: PMC10579082

20. Veitch PC, Sheehan MC, Holmes JH, Doolan T, Wallace A. Barriers to the use of urban medical services by rural and remote area households. Aust J Rural Health. 1996 May;4(2):104–110. PMID: 9437131

21. Oliveira Trindade B, Brandão GR, Bueno Motter S. Geospatial Analysis of Accessibility to Surgical Care, a Brazilian Local Perspective. World J Surg. 2023 Apr;47(4):887–894. PMID: 36645425

22. Qian Y, Gao J, Zhou Z, Yan J, Xu Y, Yang X, Li Y. An equity analysis of health examination service utilization by women from underdeveloped areas in western China. PLoS One. 2017;12(10):e0186837. PMCID: PMC5655443

23. Wilcox LS, Mosher WD. Factors associated with obtaining health screening among women of reproductive age. Public Health Rep. 1993;108(1):76–86. PMCID: PMC1403334

24. Brunner-Ziegler S, Rieder A, Stein KV, Koppensteiner R, Hoffmann K, Dorner TE. Predictors of participation in preventive health examinations in Austria. BMC Public Health. 2013 Dec 5;13:1138. PMCID: PMC3866300

25. Chang WC, Lan TH, Ho WC, Lan TY. Factors affecting the use of health examinations by the elderly in Taiwan. Archives of Gerontology and Geriatrics. 2010 Feb 1;50:S11–S16.

26. Larson S, Correa-de-Araujo R. Preventive health examinations: A comparison along the rural–urban continuum. Women’s Health Issues. 2006 Mar 1;16(2):80–88.

27. Hashemzadeh M, Shariati M, Mohammad Nazari A, Keramat A. Childbearing intention and its associated factors: A systematic review. Nurs Open. 2021 Mar 11;8(5):2354–2368. PMCID: PMC8363403

28. Kolk M. The relationship between life-course accumulated income and childbearing of Swedish men and women born 1940-70. Popul Stud (Camb). 2023 Jul;77(2):197–215. PMID: 36377741

29. Gold R, Connell FA, Heagerty P, Bezruchka S, Davis R, Cawthon ML. Income inequality and pregnancy spacing. Social Science & Medicine. 2004 Sep 1;59(6):1117–1126.

30. Lazzari E, Mogi R, Canudas-Romo V. Educational composition and parity contribution to completed cohort fertility change in low-fertility settings. Popul Stud (Camb). 2021 Jul;75(2):153–167. PMID: 33780319

31. Ayele AD, Belay HG, Kassa BG, Worke MD. Knowledge and utilisation of preconception care and associated factors among women in Ethiopia: systematic review and meta-analysis. Reprod Health. 2021 Apr 15;18(1):78. PMCID: PMC8048176

32. Munthali M, Chiumia IK, Mandiwa C, Mwale S. Knowledge and perceptions of preconception care among health workers and women of reproductive age in Mzuzu City, Malawi: a cross-sectional study. Reprod Health. 2021 Nov 14;18:229. PMCID: PMC8591898

33. Daly MP, White J, Sanders J, Kipping RR. Women’s knowledge, attitudes and views of preconception health and intervention delivery methods: a cross-sectional survey. BMC Pregnancy Childbirth. 2022 Sep 24;22:729. PMCID: PMC9508727

34. Clark AD, Mager NAD. “Nobody talks about it”: Preconception health and care among women in the rural, Midwestern United States. Womens Health (Lond). 2022 May 13;18:17455057221097563. PMCID: PMC9109168

35. Wang W, Zhang Y, Lin B, Mei Y, Ping Z, Zhang Z. The Urban-Rural Disparity in the Status and Risk Factors of Health Literacy: A Cross-Sectional Survey in Central China. Int J Environ Res Public Health. 2020 May 29;17(11):3848. PMCID: PMC7312746

36. Dennis CL, Prioreschi A, Birken CS, Brennenstuhl S, Brown HK, Bell RC, Marini F, Wrottesley SV. Predictors of preconception health knowledge among Canadian women: A nationwide cross-sectional study. J Clin Nurs. 2023 Aug;32(15–16):4843–4851. PMID: 36460481

37. Al-Akour NA, Sou’Ub R, Mohammad K, Zayed F. Awareness of preconception care among women and men: A study from Jordan. J Obstet Gynaecol. 2015 Apr;35(3):246–250. PMID: 25265237

38. Chokshi DA. Income, Poverty, and Health Inequality. JAMA. 2018 Apr 3;319(13):1312–1313. PMID: 29614168

39. Kondo N, Sembajwe G, Kawachi I, van Dam RM, Subramanian SV, Yamagata Z. Income inequality, mortality, and self rated health: meta-analysis of multilevel studies. BMJ. 2009 Nov 10;339:b4471. PMCID: PMC2776131

40. Li C, Tang C. Income-related health inequality among rural residents in western China. Front Public Health. 2022;10:1065808. PMCID: PMC9797679

41. Bor J, Cohen GH, Galea S. Population health in an era of rising income inequality: USA, 1980-2015. Lancet. 2017 Apr 8;389(10077):1475–1490. PMID: 28402829

42. Korn AR, Walsh-Bailey C, Correa-Mendez M, DelNero P, Pilar M, Sandler B, Brownson RC, Emmons KM, Oh AY. Social determinants of health and US cancer screening interventions: A systematic review. CA Cancer J Clin. 2023;73(5):461–479. PMCID: PMC10529377

43. Montez JK, Friedman EM. Educational attainment and adult health: under what conditions is the association causal? Soc Sci Med. 2015 Feb;127:1–7. PMID: 25557617

44. Luo D, Du J, Wang P, Yang W. Urban-rural comparisons in health risk factor, health status and outcomes in Tianjin, China: A cross-sectional survey (2009-2013). Aust J Rural Health. 2019 Dec;27(6):535–541. PMID: 31614059

45. Li Y, Wang L, Jiang Y, Zhang M, Wang L. Risk factors for noncommunicable chronic diseases in women in China: surveillance efforts. Bull World Health Organ. 2013 Sep 1;91(9):650–660. PMCID: PMC3790222

46. Bearak J, Popinchalk A, Ganatra B, Moller AB, Tunçalp Ö, Beavin C, Kwok L, Alkema L. Unintended pregnancy and abortion by income, region, and the legal status of abortion: estimates from a comprehensive model for 1990-2019. Lancet Glob Health. 2020 Sep;8(9):e1152–e1161. PMID: 32710833

47. Ampt FH, Willenberg L, Agius PA, Chersich M, Luchters S, Lim MSC. Incidence of unintended pregnancy among female sex workers in low-income and middle-income countries: a systematic review and meta-analysis. BMJ Open. 2018 Sep 17;8(9):e021779. PMCID: PMC6144321

48. Tran TP, Liu JJ. Interrelationships Between Pregnancy Intention, Antenatal Care, and Sociodemographic Factors: Analysis of a Nationwide Population-Based Cross-Sectional Study in Vietnam. Matern Child Health J. 2023 Jan;27(1):142–150. PMID: 36352284

49. Lelis C de F, Prietsch SOM, Cesar JA. Unplanned pregnancy in the extreme South of Brazil: prevalence, trends, and associated factors. Cien Saude Colet. 2024 May;29(5):e11122023. PMID: 38747772

50. Alene M, Yismaw L, Berelie Y, Kassie B, Yeshambel R, Assemie MA. Prevalence and determinants of unintended pregnancy in Ethiopia: A systematic review and meta-analysis of observational studies. PLoS One. 2020 Apr 7;15(4):e0231012. PMCID: PMC7138300

51. Sutton A, Lichter DT, Sassler S. Rural–Urban Disparities in Pregnancy Intentions, Births, and Abortions Among US Adolescent and Young Women, 1995–2017. Am J Public Health. 2019 Dec;109(12):1762–1769. PMCID: PMC6836770

52. Che Y, Li Y, Gu X, Jiang L, Zhou Y, Hu X, Jiang L, Cheng T, Dong X, Huang X, Luo Y, Lv W, Qiao G, Song J, Xia W, Zhang L, Zhou Y, Zhang Y. Contraception, unintended pregnancy, and induced abortion within 24 months of delivery in China: a retrospective cohort study. Contraception. 2021 Mar 1;103(3):144–150.

53. Azar A, Maldonado L, Castillo JC, Atria J. Income, egalitarianism and attitudes towards healthcare policy: a study on public attitudes in 29 countries. Public Health. 2018 Jan 1;154:59–69.

54. Demeke M, Yetwale F, Mulaw Z, Yehualashet D, Gashaw A, Agegn Mengistie B. Knowledge and attitude towards preconception care and associated factors among women of reproductive age with chronic disease in Amhara region referral hospitals, Ethiopia, 2022. BMC Womens Health. 2024 Mar 19;24(1):184. PMCID: PMC10949722

55. Okemo JK, Kamya D, Mwaniki AM, Temmerman M. Determinants of preconception care among pregnant women in an urban and a rural health facility in Kenya: a qualitative study. BMC Pregnancy Childbirth. 2021 Nov 8;21(1):752. PMCID: PMC8573977

56. Yang W, Zhou L, Shi C, Ye D, Yan X, Zhang Y, Liao Y, Pan L. [Status and related factors on knowledge, attitude and practice of adults environmental health in four cities in 2021]. Wei Sheng Yan Jiu. 2023 Jan;52(1):95–99. PMID: 36750335

**Supplementary Table 3: Model parameters**

| Model (basic architecture)/Parameters | Range explored | Final selected value of Parameters | | | | | | | | |
| --- | --- | --- | --- | --- | --- | --- | --- | --- | --- | --- |
|  |  | Total | With University Education | Withou University Education | Urban | Rural | Accepted APMCH | Unaccepted APMCH | Planned pregnancy | Unplanned pregnancy |
| RandomForest: Best cross-validation score (AUC) |  | 0.858561652 | 0.834411082 | 0.873677215 | 0.841968125 | 0.859161545 | 0.799513904 | 0.909186176 | 0.792228629 | 0.830911762 |
| n_estimators | (10L, 1000L) | 758L | 558L | 982L | 758L | 758L | 758L | 1000L | 758L | 497L |
| max_depth | (1L, 30L) | 10L | 7L | 24L | 10L | 10L | 10L | 30L | 10L | 4L |
| min_samples_split | (2L, 50L) | 48L | 4L | 35L | 48L | 48L | 48L | 2L | 48L | 10L |
| max_features | (1L, 21L) | 8L | 7L | 4L | 8L | 8L | 8L | 17L | 8L | 15L |
| min_samples_leaf | (1L, 50L) | 28L | 50L | 12L | 28L | 28L | 28L | 21L | 28L | 22L |
| XGBoost: Best cross-validation score (AUC) |  | 0.857700154 | 0.83300331 | 0.874268403 | 0.840123642 | 0.85966878 | 0.79943565 | 0.908053192 | 0.788228905 | 0.828667265 |
| eta | (0.001, 0.3) | 0.221380266 | 0.226546328 | 0.112938177 | 0.104665199 | 0.008000779 | 0.138376513 | 0.015549098 | 0.216220748 | 0.03 |
| max_depth | (1L, 30L) | 1L | 3L | 1L | 1L | 5L | 1L | 3L | 1L | 22L |
| n_estimators | (10L, 500L) | 445L | 11L | 210L | 258L | 500L | 433L | 260L | 499L | 172L |
| min_child_weight | (0L, 20L) | 21L | 20L | 3L | 20L | 20L | 14L | 20L | 7L | 12L |
| subsample | (0.5, 1) | 0.970751929 | 0.587028853 | 0.968909381 | 0.59223233 | 0.5 | 1 | 0.587087945 | 0.933271633 | 0.835870376 |
| colsample_bytree | (0.5, 1) | 0.958829636 | 0.88923045 | 0.759726872 | 0.537309837 | 0.781413467 | 0.810754513 | 0.886520777 | 1 | 0.856129461 |
| reg_alpha | (0, 1) | 0.714811821 | 0.891513404 | 1 | 0.70953415 | 0.975517389 | 0.98954143 | 0.208837642 | 1 | 0.002953033 |
| reg_lambda | (0, 1) | 0.390138954 | 0.124513373 | 0.09831455 | 0.823335051 | 0.988737323 | 0.392564475 | 0.676653534 | 0.86476493 | 0.843809639 |

**Supplementary Table 4: Model performance metrics**

| **Model** | **AUROC** | **Accuracy** | **Sensitivity** | **Specificity** | **Precision** | **F1-score** |
| --- | --- | --- | --- | --- | --- | --- |
| Logistic | 0.856  (0.848, 0.864) | 0.782  (0.773, 0.790) | 0.761  (0.747, 0.774) | 0.797  (0.785, 0.808) | 0.734  (0.720, 0.748) | 0.747  (0.736, 0.758) |
| RandomForest | 0.908  (0.902, 0.914) | 0.828  (0.820, 0.836) | 0.824  (0.813, 0.836) | 0.830  (0.820, 0.841) | 0.781  (0.768, 0.794) | 0.802  (0.792, 0.812) |
| XGBooost | 0.867  (0.859, 0.874) | 0.792  (0.783, 0.800) | 0.789  (0.776, 0.802) | 0.793  (0.782, 0.804) | 0.738  (0.724, 0.752) | 0.763  (0.752, 0.773) |

**Supplementary Table 5: Subgroup analysis of Logistic**

| Variables | With University Education | Without University Education | Urban | Rural | Accepted APMCH | Unaccepted APMCH | Planned pregnancy | Unplanned pregnancy |
| --- | --- | --- | --- | --- | --- | --- | --- | --- |
| Income | 3.10% | 1.40% | 1.10% | 1.82% | 1.64% | 4.81% | 6.25% | 1.31% |
| Educational Attainment | NA | NA | 23.12% | 5.83% | 2.47% | 17.34% | 6.97% | 12.51% |
| Census Registration (Urban-Rural) | 3.24% | 0.00% | NA | NA | 2.28% | 4.58% | 4.69% | 4.32% |
| Regions | 2.63% | 0.09% | 1.62% | 0.32% | 2.39% | 1.82% | 0.00% | 0.05% |
| Distance to Nearest MCH | 0.63% | 3.43% | 0.42% | 2.05% | 0.06% | 0.40% | 0.51% | 4.15% |
| Annual PCC Promotion at Local MCH Institutions | 9.24% | 20.95% | 7.09% | 14.36% | NA | NA | 10.91% | 20.53% |
| Annual Physical Examination | 0.43% | 4.54% | 0.06% | 3.03% | 3.32% | 0.00% | 0.88% | 3.99% |
| **CIRCUMSTANCE** | 19.28% | 30.40% | 33.40% | 27.41% | 12.17% | 28.96% | 30.21% | 46.86% |
| Age | 1.51% | 5.91% | 1.74% | 2.61% | 4.29% | 1.33% | 1.15% | 0.19% |
| Parity | 4.14% | 1.56% | 5.42% | 1.02% | 3.42% | 3.08% | 5.42% | 2.03% |
| Knowledge of PCC |  |  |  |  |  |  |  |  |
| *Knowledge of PCC Services* | 43.32% | 32.53% | 34.61% | 36.17% | 44.68% | 34.26% | 47.36% | 44.85% |
| *Knowledge of PCC Policy* | 0.00% | 2.19% | 0.01% | 2.23% | 1.60% | 0.00% | 0.39% | 0.05% |
| Health Status |  |  |  |  |  |  |  |  |
| *Common infectious diseases, chronic diseases and other disease* | 0.17% | 0.00% | 0.11% | 0.03% | 0.00% | 0.24% | 0.08% | 0.18% |
| *Adverse pregnancy outcomes* | 0.05% | 0.21% | 0.00% | 0.00% | 0.00% | 0.00% | 0.00% | 0.00% |
| *Reproductive system diseases* | 1.65% | 1.46% | 1.02% | 1.40% | 1.10% | 2.38% | 2.70% | 0.70% |
| Pregnancy Intention | 19.00% | 21.86% | 15.42% | 24.36% | 23.44% | 21.47% | NA | NA |
| Attitude towards PCC | 10.88% | 3.88% | 8.28% | 4.77% | 9.29% | 8.27% | 12.68% | 5.14% |
| **EFFORT** | 80.72% | 69.60% | 66.60% | 72.59% | 87.83% | 71.04% | 69.79% | 53.14% |

**Supplementary Table 6: Subgroup analysis of RandomForest**

| Variables | With University Education | Without University Education | Urban | Rural | Accepted APMCH | Unaccepted APMCH | Planned pregnancy | Unplanned pregnancy |
| --- | --- | --- | --- | --- | --- | --- | --- | --- |
| Income | 0.63% | 0.67% | 0.95% | 0.58% | 0.50% | 1.36% | 0.74% | 0.84% |
| Educational Attainment | NA | NA | 0.75% | 0.24% | 0.29% | 0.39% | 0.41% | 0.54% |
| Census Registration (Urban-Rural) | 0.13% | 0.05% | NA | NA | 0.09% | 0.06% | 0.16% | 0.11% |
| Regions | 0.40% | 0.05% | 0.24% | 0.07% | 0.15% | 0.05% | 0.09% | 0.66% |
| Distance to Nearest MCH | 0.12% | 0.62% | 0.11% | 0.76% | 0.78% | 0.88% | 0.38% | 0.47% |
| Annual PCC Promotion at Local MCH Institutions | 1.75% | 8.17% | 0.86% | 2.96% | NA | NA | 3.72% | 11.87% |
| Annual Physical Examination | 2.23% | 6.14% | 2.18% | 4.16% | 5.17% | 1.36% | 1.52% | 34.35% |
| **CIRCUMSTANCE** | 5.26% | 15.70% | 5.09% | 8.77% | 6.97% | 4.09% | 7.03% | 48.84% |
| Age | 0.03% | 0.71% | 0.04% | 0.09% | 0.03% | 0.09% | 0.06% | 0.12% |
| Parity | 3.60% | 0.87% | 4.05% | 0.96% | 2.20% | 2.71% | 1.56% | 3.58% |
| Knowledge of PCC |  |  |  |  |  |  |  |  |
| *Knowledge of PCC Services* | 45.34% | 44.40% | 41.90% | 50.87% | 48.12% | 49.34% | 63.17% | 33.10% |
| *Knowledge of PCC Policy* | 2.64% | 7.26% | 1.35% | 3.80% | 2.95% | 1.09% | 2.24% | 1.38% |
| Health Status |  |  |  |  |  |  |  |  |
| *Common infectious diseases, chronic diseases and other disease* | 0.24% | 1.47% | 0.50% | 0.25% | 0.31% | 0.35% | 0.32% | 0.60% |
| *Adverse pregnancy outcomes* | 0.29% | 0.73% | 0.22% | 0.21% | 0.34% | 0.19% | 0.25% | 0.18% |
| *Reproductive system diseases* | 1.95% | 1.27% | 0.74% | 1.59% | 1.68% | 5.03% | 3.38% | 0.58% |
| Pregnancy Intention | 23.80% | 18.02% | 25.74% | 26.69% | 28.00% | 16.98% | NA | NA |
| Attitude towards PCC | 16.85% | 9.57% | 20.38% | 6.77% | 9.39% | 20.13% | 22.01% | 11.63% |
| **EFFORT** | 94.74% | 84.30% | 94.91% | 91.23% | 93.03% | 95.91% | 92.97% | 51.16% |

**Supplementary Table 7: Subgroup analysis of XGBoost**

| Variables | With University Education | Without University Education | Urban | Rural | Accepted APMCH | Unaccepted APMCH | Planned pregnancy | Unplanned pregnancy |
| --- | --- | --- | --- | --- | --- | --- | --- | --- |
| Income | 0.51% | 0.77% | 1.87% | 1.28% | 0.50% | 1.35% | 1.28% | 5.19% |
| Educational Attainment | NA | NA | 3.23% | 0.39% | 0.69% | 1.41% | 2.53% | 3.68% |
| Census Registration (Urban-Rural) | 0.74% | 0.00% | NA | NA | 0.00% | 0.00% | 0.58% | 0.56% |
| Regions | 0.00% | 0.00% | 0.00% | 0.15% | 0.21% | 0.15% | 0.33% | 1.32% |
| Distance to Nearest MCH | 0.95% | 0.00% | 0.25% | 1.16% | 1.30% | 0.68% | 0.33% | 5.56% |
| Annual PCC Promotion at Local MCH Institutions | 0.00% | 13.20% | 2.76% | 3.27% | NA | NA | 4.40% | 8.41% |
| Annual Physical Examination | 3.26% | 9.42% | 3.44% | 6.37% | 5.64% | 1.10% | 2.64% | 7.94% |
| **CIRCUMSTANCE** | 5.47% | 23.39% | 11.54% | 12.63% | 8.34% | 4.69% | 12.08% | 32.65% |
| Age | 0.00% | 3.09% | 0.73% | 0.38% | 1.07% | 0.00% | 1.96% | 0.48% |
| Parity | 3.60% | 1.11% | 9.50% | 2.42% | 6.64% | 3.60% | 7.78% | 8.51% |
| Knowledge of PCC |  |  |  |  |  |  |  |  |
| *Knowledge of PCC Services* | 48.62% | 40.56% | 35.36% | 46.54% | 45.08% | 38.06% | 52.39% | 27.62% |
| *Knowledge of PCC Policy* | 0.72% | 5.26% | 1.61% | 3.65% | 3.79% | 0.70% | 2.63% | 5.22% |
| Health Status |  |  |  |  |  |  |  |  |
| *Common infectious diseases, chronic diseases and other disease* | 0.00% | 0.58% | 0.63% | 0.51% | 1.71% | 0.39% | 1.07% | 2.17% |
| *Adverse pregnancy outcomes* | 0.00% | 0.64% | 0.51% | 0.61% | 0.81% | 0.36% | 1.03% | 2.50% |
| *Reproductive system diseases* | 0.61% | 1.63% | 3.52% | 3.18% | 3.67% | 6.55% | 4.52% | 7.47% |
| Pregnancy Intention | 21.39% | 18.89% | 18.03% | 22.71% | 18.23% | 25.94% | NA | NA |
| Attitude towards PCC | 19.60% | 4.85% | 18.57% | 7.38% | 10.66% | 19.70% | 16.54% | 13.38% |
| **EFFORT** | 94.53% | 76.61% | 88.46% | 87.37% | 91.66% | 95.31% | 87.92% | 67.35% |
